# Effects of spent mushroom substrate cultivation under greenhouse conditions on growth and fruiting of passion fruit

**DOI:** 10.3389/fpls.2026.1926220

**Published:** 2026-09-07

**Authors:** Xiuqing Wei, Xujia Tang, Liang Li, Yajun Tang, Ping Zhou, Jiahui Xu

**Affiliations:** Fruit Research Institute, Fujian Academy of Agricultural Sciences, Fuzhou, Fujian, China

**Keywords:** compound substrate, passion fruit, plant growth, rhizosphere microbiota, spent mushroom substrate

## Abstract

**Introduction:**

This study aimed to develop a sustainable spent mushroom substrate (SMS)-based compound substrate for greenhouse soilless cultivation of passion fruit (*Passiflora edulis* Sims.), to reduce SMS accumulation pollution and overcome continuous cropping obstacles in traditional soil-based systems.

**Methods:**

A greenhouse pot experiment was conducted using grafted ‘Qinmi No. 9’ passion fruit seedlings. Substrate physicochemical properties, plant growth, fruit yield and quality parameters were assessed, and rhizosphere bacterial and fungal communities of eight representative treatments were profiled by 16S rRNA and ITS amplicon sequencing.

**Results:**

Among all formulations, T4 (*Pleurotus geesteranus* SMS: seaweed mud: river sand = 3:1:1) exhibited the best overall performance. Compared with the control, T4 significantly increased single fruit weight by 8.2%, fruit number per plant by 24.8% (from 35.0 to 43.6 fruits/plant) and yield per plant by 43.1% (from 2.69 to 3.85 kg/plant). Fruit quality was also improved, with soluble solids, soluble sugar and vitamin C contents increased by 11.3%, 27.6% and 30.6%, respectively, while acidity was reduced by 19.3%. Mechanistically, T4 maintained more stable substrate bulk density, pH and electrical conductivity throughout the cultivation period, and significantly enriched beneficial rhizosphere genera (*Niastella* and *Rhodanobacter*) that were positively correlated with plant growth and fruit quality traits. In contrast, pure SMS formulations (T5, T15) exhibited higher nutrient levels but poorer plant performance, likely due to excessive electrical conductivity and unstable substrate structure.

**Conclusions:**

The T4 formulation is an optimal sustainable substrate for passion fruit, balancing physical structure, salt buffering and microbial benefits, offering a practical solution for SMS reuse.

## 1 Introduction

Passion fruit (*P. edulis* Sims.) is a perennial vine fruit crop of increasing economic importance in tropical and subtropical regions, valued for its distinctive flavor and high nutritional content. However, the expansion of passion fruit cultivation has been accompanied by persistent challenges, including continuous cropping obstacles, soil-borne diseases (stem basal rot, root rot and Fusarium wilt) and progressive soil degradation (Zhang et al., 2021; Correia et al., 2022; Lin et al., 2025; Fan et al., 2025). These constraints are particularly severe under greenhouse conditions, where continuous cultivation without rotation often leads to soil acidification, salt accumulation, nutrient imbalance and enrichment of pathogenic microorganisms in the rhizosphere (Shen et al., 2021; Yan et al., 2024). Once these problems develop, plant growth is suppressed, fruit yield and quality decline, and the economic viability of production is compromised.

To overcome soil-related limitations, soilless cultivation using formulated substrates has emerged as a promising alternative (Tuxun et al., 2025). Compared with traditional soil-based systems, substrate-based cultivation offers several advantages: improved root-zone aeration and water retention, precise nutrient management, reduced incidence of soil-borne diseases, and the possibility of recycling agricultural by-products as substrate ingredients (Gohardoust et al., 2020; Hernández et al., 2021; Tuxun et al., 2025). Among various organic materials, spent mushroom substrate (SMS) that the residual biomass generated after mushroom harvesting has attracted increasing attention as a sustainable substrate component (Hernández et al., 2021; Ma et al., 2025). SMS typically contains abundant organic matter, residual mycelia and macro-/micronutrients, making it potentially suitable for plant cultivation after proper composting (Paula et al., 2017; Narváez et al., 2021).

Nonetheless, the direct use of raw or inadequately composted SMS carries inherent risks. Uncomposted SMS may exhibit excessive electrical conductivity (EC), unstable physical structure, unbalanced carbon-to-nitrogen ratio and the potential for phytotoxic compound release, all of which can impair root development and plant growth (Li et al., 2021; Sun et al., 2023). Therefore, to transform SMS into a reliable soilless substrate ingredient, composting is necessary to stabilize its physicochemical properties, and subsequent blending with complementary materials (e.g., inorganic structural components or water-retentive organic amendments) is often required to achieve a balanced root-zone environment (Medina et al., 2009; He et al., 2024). In this context, seaweed mud (rich in polysaccharides, colloids and alginates) and river sand (providing skeletal support and drainage) have been used as functional additives to improve substrate water-holding capacity, porosity and structural stability (Espinosa-Antón et al., 2023; Singh et al., 2023).

Although SMS-based substrates have been widely applied and tested in various horticultural crops (Prasad et al., 2021; Huang et al., 2023; Chen et al., 2024; Mwangi et al., 2024; Fraszczak et al., 2025), research on their use in passion fruit cultivation is still in its early stages, with only the report by Cheng et al. (2025) available to date. That study demonstrated that co-application of an SMS-based bio-organic fertilizer with a compound fertilizer significantly increased passion fruit yield (up to 1.27 kg per plant), improved fruit quality (soluble solids content and sugar-acid ratio), and enhanced soil microbial diversity (Cheng et al., 2025). However, the mechanisms by which SMS, when used as the main cultivation substrate, affects the entire growth cycle of passion fruit remain unclear. Given that passion fruit is characterized by a long growth period and continuous flowering and fruiting, it imposes higher demands on the physicochemical stability of the substrate and sustained nutrient supply than does seedling propagation. More importantly, the rhizosphere microbiome plays a central role in nutrient cycling, pathogen suppression, and plant health regulation (Xun et al., 2021; Luo et al., 2025). Integrating the physicochemical dynamics of the substrate, plant growth performance, fruit quality formation, and shifts in rhizosphere microbial communities into a unified mechanistic framework, and elucidating how different SMS-based substrate formulations reshape the bacterial and fungal community structure in the passion fruit rhizosphere, will provide a solid theoretical basis for efficient and sustainable soilless cultivation of passion fruit.

Against this background, the present study was conducted with three main objectives: first, to evaluate the suitability of three types of composted spent mushroom substrates (SMS of *Pleurotus geesteranus*, *Auricularia heimuer*, and *Pleurotus eryngii*) as soilless substrate ingredients for passion fruit production; second, to compare the effects of 15 compound substrate formulations (varying in SMS type and blending ratio with seaweed mud and river sand) on substrate physicochemical properties, plant growth, fruit yield and quality; and third, to examine, through high-throughput amplicon sequencing, how representative substrate formulations influence the composition and diversity of rhizosphere bacterial and fungal communities, and to link these microbial shifts to plant performance. By addressing these objectives, this study aims to provide a scientific basis for SMS valorization and the development of a tailored, sustainable substrate for greenhouse passion fruit cultivation.

## 2 Materials and methods

### 2.1 Plant materials and treatments

The experiment was conducted in a plastic greenhouse at the Institute of Digital Agriculture, Fujian Academy of Agricultural Sciences, Fuzhou, Fujian Province, China (26°07′N, 119°22′E). Grafted seedlings of passion fruit (*P. edulis* Sims.) cultivar ‘Qinmi No. 9’ were used as the test material.

Three types of spent mushroom substrate (SMS of *P. geesteranus*, *A. heimuer* and *P. eryngii*) were obtained from Fujian Wanyi Agricultural Development Co., Ltd. (Zhangzhou, China). Each SMS was composted with pre-composted pig manure at a 20:1 (SMS: manure) mass ratio under aerobic conditions. The composting process lasted until the pile temperature stabilized near ambient temperature. After composting, each SMS was air-dried, crushed and sieved through an 8 mm screen.

Composted SMS was then blended with seaweed mud and river sand (particle size ~6 mm) at different volume ratios (**Table 1**). A total of 16 formulations were prepared, including 15 SMS-based compound substrates and a conventional soil-based control (CK: yellow soil: seaweed mud: river sand = 2:1:1). Each treatment was replicated in seven non-woven substrate bags (50 cm diameter × 40 cm height; approximately 60 L per bag; two plants per bag). The experiment followed a completely randomized design. All treatments received the same fertilizer regime: a slow-release compound fertilizer (N:P_2_O_5_:K_2_O = 15:15:15, Sinofert Holdings Limited, China) at 50 g per plant every 1~2 months, and water-soluble fertilizers (N:P_2_O_5_:K_2_O = 20%:20%:20% or N:P_2_O_5_:K_2_O = 15:5:30, Sinofert Holdings Limited, China) at 100~300 × dilution every 7~10 days.

**Table 1:** Different formulations of cultivation substrates for *P. edulis*.

SMS type	Treatment	Ratio (V:V:V)			SMS type	Treatment	Ratio (V:V:V)		
		SMS	Seaweed mud	River sand			SMS	Seaweed mud	River sand
*Pleurotus geesteranus*	T1	1	2	2	*Pleurotus eryngii*	T11	1	2	2
	T2	1	1	1		T12	1	1	1
	T3	2	1	1		T13	2	1	1
	T4	3	1	1		T14	3	1	1
	T5	1	0	0		T15	1	0	0
*Auricularia heimuer*	T6	1	2	2					
	T7	1	1	1					
	T8	2	1	1					
	T9	3	1	1					
	T10	1	0	0					

### 2.2 Substrate physicochemical analysis

The substrate was packed into a cup of known volume (V, cm³) and initial weight (W_0_, g), with the substrate overfilling the cup, and the total weight (W_1_) was recorded. The cup mouth was covered with two layers of gauze secured with a rubber band, and the cup was then placed into a larger container. Water was added to the container until the cup was completely submerged. After soaking for 24 h, the cup was removed, the rubber band was cut off, and the cup with the saturated substrate (without the gauze) was weighed (W_2_). The wet gauze was weighed separately (W_3_). The cup was then inverted on filter paper to allow excess water to drain until no further water drained, and the final weight (W_4_) was recorded. The following formulas were used for calculation:


$$\text{Bulk density }(\text{g}\cdot {\text{cm}}^{-3})=({\text{W}}_{1}–{\text{W}}_{0})/\text{V}$$

$$\text{Total porosity }(\%)=({\text{W}}_{2}–{\text{W}}_{1})/\text{V}\times 100$$

$$\text{Aeration porosity }(\%)=({\text{W}}_{2}+{\text{W}}_{3}–{\text{W}}_{4})/\text{V}\times 100$$

For pH and EC, samples were extracted with deionized water (1:5, w/v), shaken at 240 rpm for 15 min, centrifuged, and the supernatant measured using a pH meter (PHS-25) and a conductivity meter (DDSJ-308A, both Shanghai Leici) (Zhao et al., 2022; Goswami et al., 2024). Soil organic matter content was determined using the titration method (potassium dichromate oxidation) (Walkley and Black, 1934) with a heavy metal digestion apparatus (SH230, Shandong Haian Scientific Instrument Co., Ltd.) and a titration setup (Deng et al., 2020). Soil total nitrogen (N) and alkali-hydrolyzable nitrogen were determined by the Kjeldahl method (K1160, Shandong Haian Scientific Instrument Co., Ltd.) (Bremner, 1996) and the diffusion absorption method (Cornfield, 1960), respectively. Total phosphorus (P) was measured by the acid digestion-molybdenum antimony colorimetric method (Olsen and Sommers, 1982), and available phosphorus by the hydrochloric acid-ammonium fluoride extraction-molybdenum antimony colorimetric method (Bray and Kurtz, 1945); both were analyzed using a spectrophotometer (T6 New Century, Beijing Purkinje General Instrument Co., Ltd.). Total potassium (K) and available potassium were determined by nitric-perchloric-hydrofluoric acid digestion combined with inductively coupled plasma optical emission spectrometry (ICP-OES) (AA-3300F, Shanghai Yuanxi Instrument Co., Ltd.) (Rao et al., 2011) and by the ammonium acetate extraction-flame photometry method (AP1302, Shanghai Aopu Analytical Instrument Co., Ltd.), respectively (Zheng et al., 2024; Cheng et al., 2025).

### 2.3 Plant growth, yield and fruit quality measurements

Measurements were scheduled according to the developmental phases of passion fruit. The 0-50 d period was designated as the vegetative stage, during which vegetative growth parameters were intensively measured. From 50 to 200 d, plants entered the reproductive stage. During this period, leaf nutrient status was continuously monitored, and fruit quality and yield were assessed at fruit maturity.

Plant growth indicators (height, stem diameter and leaf number) were recorded at 0, 15, 30, 35, 40, 45 and 50 days after planting. Height (cm) from the graft union to the main vine tip was measured with a tape measure, and stem diameter (mm) at 1 cm above the graft union with a vernier caliper; fully expanded functional leaves were counted. At 0 and 50 days, three plants per treatment were randomly sampled. Roots were carefully removed, rinsed, and surface-dried. Root length (mm) from stem base to the longest root tip was measured with a ruler, and root volume (cm³) by water displacement. Each plant was separated into aboveground and belowground parts for fresh weight (g), then oven-dried at 105 °C for 30 min (deactivation) followed by 75 °C to constant weight for dry weight (g). At 50 days (after transplant recovery and during stable, vigorous vegetative growth), on a sunny morning (9:00~11:00 a.m.), three uniform plants per treatment were selected. Net photosynthetic rate (Pn), transpiration rate (Tr), stomatal conductance (Gs), and intercellular CO_2_ concentration (Ci) were measured using a LI-6400 portable photosynthesis system. The 10th fully expanded functional leaf (Ferreira et al., 2022) from the apex of the main vine was selected for measurement under prevailing greenhouse conditions, with data recorded after gas-exchange readings had stabilized. Leaf samples were collected at 0, 50, 100, 150 and 200 days after planting: for the first three times, the 10th leaf from the main vine tip; for the last two, the 10th leaf from the tip of the nearest secondary or tertiary vine. These samples were analyzed for: photosynthetic pigments (extracted with anhydrous ethanol in darkness; absorbance at 665, 649, and 470 nm; chlorophyll a, chlorophyll b and total chlorophyll calculated in mg·g^−^¹ FW) (Lichtenthaler and Wellburn, 1983); soluble sugars (anthrone colorimetric method after hot water extraction, glucose standard curve) (Yemm and Willis, 1954); soluble protein (Coomassie Brilliant Blue G-250 method, bovine serum albumin standard curve) (Bradford, 1976); total nitrogen (Kjeldahl method: H_2_SO_4_ digestion, alkaline distillation, NH_3_ trapped in boric acid, titration with standard acid) (Motsara and Roy, 2008); total phosphorus and total potassium (acid digestion followed by ICP-OES) (Donohue and Aho, 1992). For destructive sampling parameters (root traits and biomass), three cultivation bags were randomly selected from seven, and one plant was sampled from each bag at 50 days after planting, with each bag considered as a single independent replicate (n = 3). For non-destructive parameters (plant height, stem diameter, and leaf number), measurements were recorded from both plants in each remaining bag, and the average value was used as the representative replicate for that bag.

At fruit maturity (200 d), 10 as biological replicates uniform, undamaged fruits free from pests and diseases were randomly selected per treatment to assess external quality (single fruit weight, longitudinal diameter, transverse diameter, fruit shape index) and internal quality (soluble solids, acidity, soluble sugars, soluble protein, edible rate, and vitamin C). Single fruit weight was measured with an electronic balance. Longitudinal diameter (apex-to-base length) and transverse diameter (equatorial width) were measured with a vernier caliper, and the fruit shape index was calculated as their ratio. Total soluble solids (TSS) and acidity were measured using a digital refractometer (PAL-1, ATAGO Co., Ltd., Tokyo, Japan). Soluble sugars were quantified by the anthrone colorimetric method, and soluble protein by the Coomassie Brilliant Blue G-250 method. Edible rate = (edible portion weight/whole fruit weight) × 100%. Vitamin C was measured by the 2,6-dichloroindophenol titration method. Yield indicators included the number of fruits per plant (summed over three harvests) and fruit yield per plant (cumulative mass per plant).

### 2.4 Rhizosphere microbiota analysis

Based on the comprehensive performance across substrate properties, plant growth and fruit traits, eight representative treatments were selected for rhizosphere microbial analysis: CK, T3, T4, T5, T6, T8, T13 and T15. At 200 days after transplanting, rhizosphere substrate samples were collected from three independent biological replicates per treatment by gently shaking off loosely attached substrate from the roots and retaining the tightly adhering fraction. Total DNA was extracted from each replicate sample, and the bacterial 16S rRNA gene full-length region and fungal ITS region were amplified and sequenced on an Oxford Nanopore Technologies platform. Raw Nanopore reads were base-called and demultiplexed with Dorado, followed by adapter/barcode removal with Porechop, quality filtering (Q < 10) with NanoFilt, and primer trimming with Cutadapt (Martin, 2011). For taxonomic annotation, cleaned reads were aligned against Silva (16S) (Quast et al., 2013) and UNITE (ITS) (Abarenkov et al., 2024) databases using LAST, with best-hit alignments used to construct a feature table. Features with total abundance ≤ 1 were removed, and the table was rarefied to the minimum sequencing depth. Community composition was analyzed at taxonomic levels from phylum to species, and visualized using bar plots, heatmaps, and shared/unique feature analysis. α-Diversity was estimated using the Chao1 and Shannon indices, while β-diversity was assessed by principal coordinate analysis (PCoA) and analysis of similarities (ANOSIM) based on Bray-Curtis distances. Linear discriminant analysis effect size (LEfSe) (Segata et al., 2011) was applied to identify differentially abundant taxa among treatments. Spearman correlation analysis and Mantel tests were used to explore relationships among substrate properties, microbial community structure and plant traits.

### 2.5 Statistical analysis

Data were compiled using Microsoft Excel. Before analysis, normality (Shapiro-Wilk test) and homogeneity of variances (Levene’s test) were checked to verify the assumptions of parametric ANOVA. As samples at different growth stages were independently harvested, a two-way ANOVA was employed with treatment and sampling time as fixed factors, along with their interaction term. Where the interaction was significant, simple effects analysis was performed to compare treatments at each time point. Treatment means were separated using Duncan’s test with significance set at p< 0.05. For fruit quality and yield parameters, which were assessed at a single harvest time without a temporal dimension, one-way ANOVA was used. Pearson correlation analysis was conducted using SPSS 23.0, and the resulting P-values were corrected by the false discovery rate (FDR) method to control for multiple comparisons. Graphs were generated with Origin 2024.

## 3 Results

### 3.1 Substrate physicochemical properties

#### 3.1.1 Pre- and post-composting changes in fungal residue physicochemical and nutrient properties

Three SMSs completed aerobic composting after 3~4 heating and cooling cycles over a period of 28-32 days (**Supplementary Table 1**, **Supplementary Figure 1**). The changes in bulk density, pH, organic matter content and available N of the three SMS types converged: the former three increased, while available N content decreased. In contrast, the trends of other physicochemical indicators and nutrient contents varied. Overall, composting substantially improved the physicochemical properties of the three SMS types (**Supplementary Table 2**). After composting, *P.eryngii* SMS exhibited lower bulk density (0.25 g·cm^-3^) and higher aeration porosity (40.0%); *A.heimue* SMS showed the highest total porosity (65%) and organic matter content (5.23%); and *P.geesteranus* SMS displayed the highest total P (0.68%), total K (13.75 g·kg^-1^) and available K (6660 mg·kg^-1^). All three composted SMS had pH values in the weakly alkaline range (8.44~8.56).

#### 3.1.2 Changes in physicochemical properties of different substrates during passion fruit cultivation

**Figure 1:**
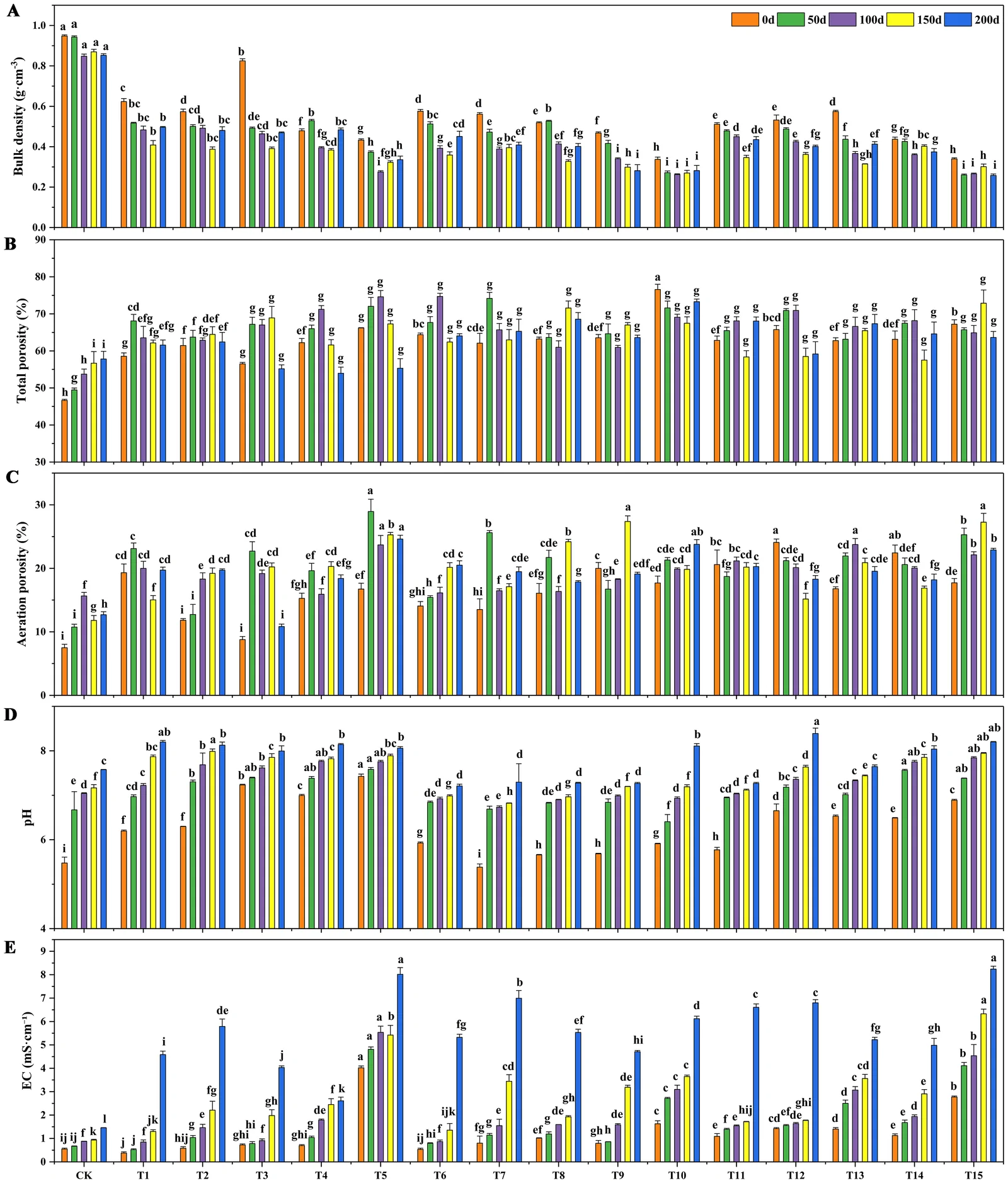
Changes in physicochemical properties of different substrate formulations at different stages from 0 to 200 d. The error bar indicates the standard errors for three replicates. Lowercase letters indicate significant differences among the 16 treatments within the same period (p < 0.05). The same below.

Using the aforementioned mature SMS, seaweed mud and river sand, 15 blended substrates were prepared at different volume ratios (**Table 1**). After blending, the physicochemical properties of each substrate improved, with overall decreases in bulk density, pH and EC, and nutrient contents concentrations decreased (**Supplementary Table 2**, **Figure 1**). The physicochemical properties and nutrient contents of all treatments were monitored throughout the passion fruit production. Across the cultivation period, the blended substrates maintained lower bulk density and higher total porosity than CK (**Figure 1**). T10 (*A. heimuer* SMS only) and T15 (*P. eryngii* SMS only) exhibited low and stable bulk density, ranging from 0.26 to 0.34 g·cm^-3^, whereas CK showed the highest bulk density (0.85~0.95 g·cm^-3^)(**Figure 1A**). Total porosity and aeration porosity were generally higher in SMS-based substrates than in CK. Notably, T4 maintained moderate-to-high aeration porosity with good stability throughout cultivation (**Figures 1B, C**). Substrate pH increased gradually across all treatments, from 5.38~7.43 to 7.21~8.39. Overall, T3, T4 and T5 (all *P. geesteranus* SMS-based) exhibited the smallest fluctuations, indicating stronger buffering capacity, whereas T10 and CK showed larger increases, and T12 exhibited the most pronounced alkalization in the later stage (**Figure 1D**). EC values increased over time in all treatments, with the smallest differences between initial and final values observed in CK and T4, which increased by only 0.91 and 1.91 mS·cm^-1^, respectively. Notably, at 200 days, the EC values of all treatments except T4 increased sharply, among which the values of T10 and T15 were significantly higher than those of other treatments at the same time point (**Figure 1E**).

#### 3.1.3 Changes in nutrient contents of different substrates during passion fruit cultivation

Organic matter content increased throughout the cultivation period, with the pure SMS treatments (T5, T10, T15) maintaining significantly higher levels than other treatments and reaching respective peaks at 200 days: 41.77%, 47.90%, and 42.65%. In contrast, CK exhibited a consistently low level, with its value at 200 days (8.51%) significantly lower than all other treatments (**Supplementary Figure 2A**). The contents of total N, total P, total K, and their corresponding available nutrients (alkali-hydrolyzable N, available P, and available K) generally increased across all treatments during cultivation, whereas CK remained at relatively low levels. For N, total N and alkali-hydrolyzable N in T5, T10 and T15 (pure SMS) were significantly higher than in other treatments, indicating strong nitrogen supply potential, while T4 showed stable total N changes and better sustained capacity (**Supplementary Figures 2B, C**). Regarding P, T5 and T15 maintained high total P and available P levels, with T5 reaching the highest available P at 200 days (1036 mg·kg^-1^); additionally, T6, T10 and T12 (all *A. heimuer* SMS-based) showed relatively high P levels in the mid-to-late stage (**Supplementary Figures 2D, E**). As for K, total K content varied dynamically over time, while available K generally increased. T1, T2 and T4 (all *P. geesteranus* SMS-based) exhibited relatively high total K levels, whereas T5 and T15 showed a marked increase in available K in the mid-to-late stage, with T15 peaking at 200 days (6214 mg·kg^-1^) (**Supplementary Figures 2F, G**).

### 3.2 Effects of different substrates on plant growth and physiological performance of passion fruit

#### 3.2.1 Vegetative growth

**Figure 2:**
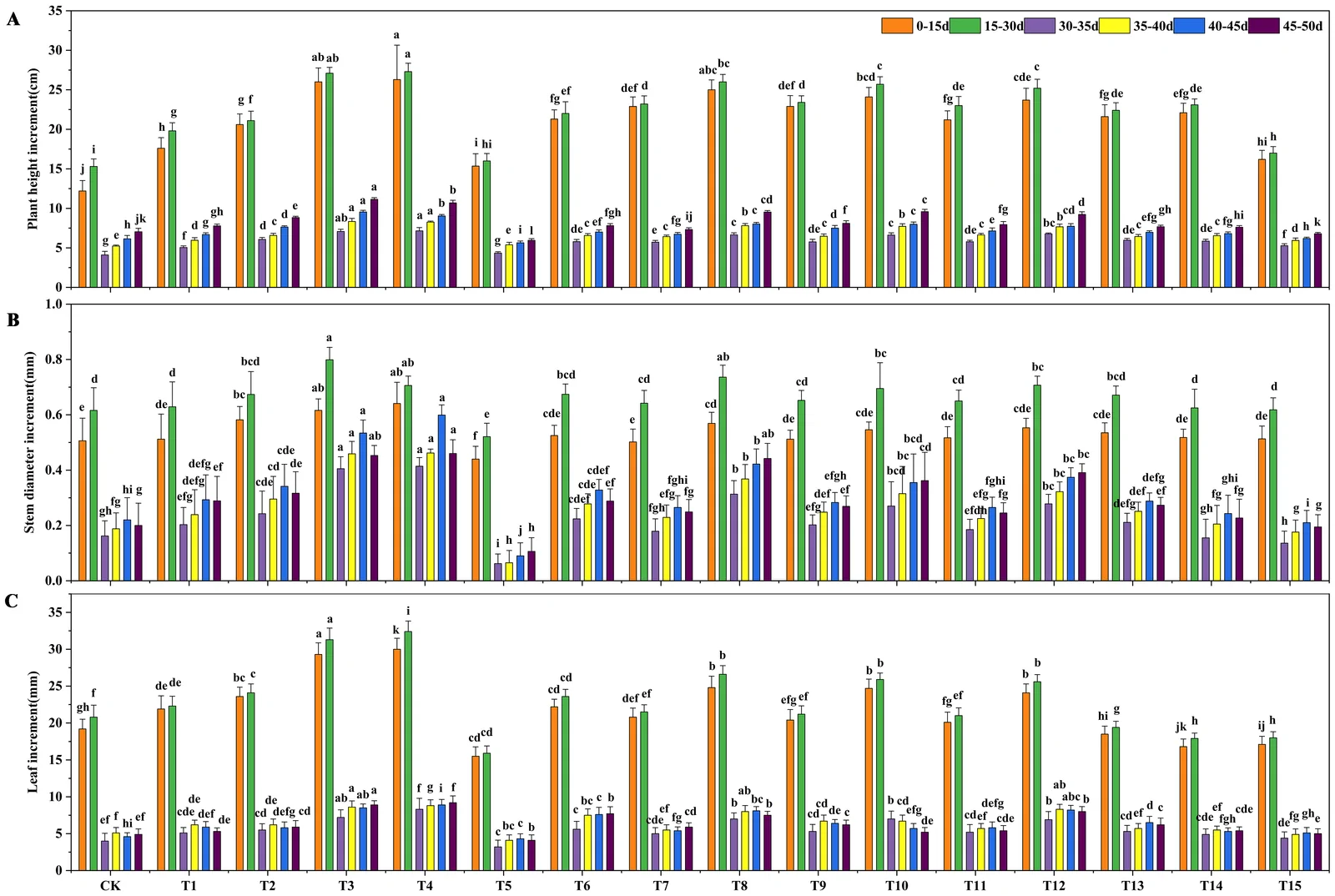
Effects of different substrate treatments on the vegetative growth of passion fruit (0~50 days). **(A)** Changes in plant height increment; **(B)** Changes in stem diameter; **(C)** Changes in leaf increment. The error bar indicates the standard errors for three replicates.

The study showed that different substrate treatments had significant effects on the vegetative growth of passion fruit, with T3 and T4 (both *P. geesteranus* SMS-based) performing the best. During 0~30 days after planting, plant height, stem diameter, and leaf increment increased rapidly across all treatments, with the highest growth rates observed in the 15~30 day period. T3 and T4 exhibited the greatest increases in plant height (26.0~27.3 cm), stem diameter (0.71~0.80 mm), and leaf number (29.3~32.4) during this stage, and these increments were significantly higher than those of CK. Although the growth of all indicators generally declined from 30 to 50 days after planting, T3 and T4 still maintained relatively high levels: T3 achieved a plant height increase of 11.14 cm at 45~50 days; T4 showed a stem diameter increase of 0.60 mm at 40~45 days and a leaf increment of 9.2 at 45~50 days. In contrast, CK, T5 and T15 consistently exhibited lower values throughout all periods. In conclusion, T3 and T4 were more effective in promoting early-stage plant height elongation, stem thickening, and leaf emergence in passion fruit (**Figure 2**).

#### 3.2.2 Root system and biomass accumulation

For root growth, T4 had the highest root length increment (96.75 mm), and T3 the greatest root volume increment (110.57 cm³), both exceeding CK. Conversely, T5 and T15 exhibited poorer root growth. For biomass accumulation, T3 achieved the highest increments in belowground fresh weight (31.84 g), aboveground dry weight (210.03 g), and belowground dry weight (19.68 g), favoring dry matter accumulation. T4 showed relatively high increments in aboveground (399.79 g) and belowgrou -nd (24.51 g) fresh weight, promoting overall biomass formation. T9 had the highest aboveground fresh weight increment (418.29 g), while T12 and T13 also showed high aboveground dry weight increments. In comparison, T15 had consistently lower values across all indicators, an aboveground fresh weight increment of only 165.11 g (**Supplementary Table 3**). Overall, T3 and T4 were optimal: T3 favored belowground growth and dry matter accumulation, whereas T4 promoted aboveground growt -h and overall biomass formation.

#### 3.2.3 Photosynthetic characteristics of leaves

Different substrate treatments significantly affected the chlorophyll content and photosynthetic characteristics of passion fruit leaves. Chlorophyll content initially increased and then decreased across treatments, peaking at 100 days after planting. T4 performed best at all stages (reaching 3.06 mg·g^-1^ FW at 100 days), with T2 and T3 also maintaining relatively high levels, whereas CK, T5 and T15 showed consistently lower values (**Supplementary Figure 3A**). Regarding photosynthetic traits, T4 had the highest net photosynthetic rate (22.31 μmol·m^-2^·s^-1^) and stomatal conductance (0.91 mol H_2_O·m^-2^·s^-1^); T12 exhibited the highest transpiration rate (1.56 mmol H_2_O·m^-2^·s^-1^); and T14 had the highest intercellular CO_2_ concentration (736.87 μmol CO_2_·mol^-1^). The CK consistently showed lower values across all indicators (**Supplementary Figure 3B-E**). Thus, T4 is the most effective treatment for enhancing chlorophyll accumulation, photosynthetic capacity, and gas exchange efficiency in passion fruit leaves.

#### 3.2.4 Nutritional characteristics of leaves

**Figure 3:**
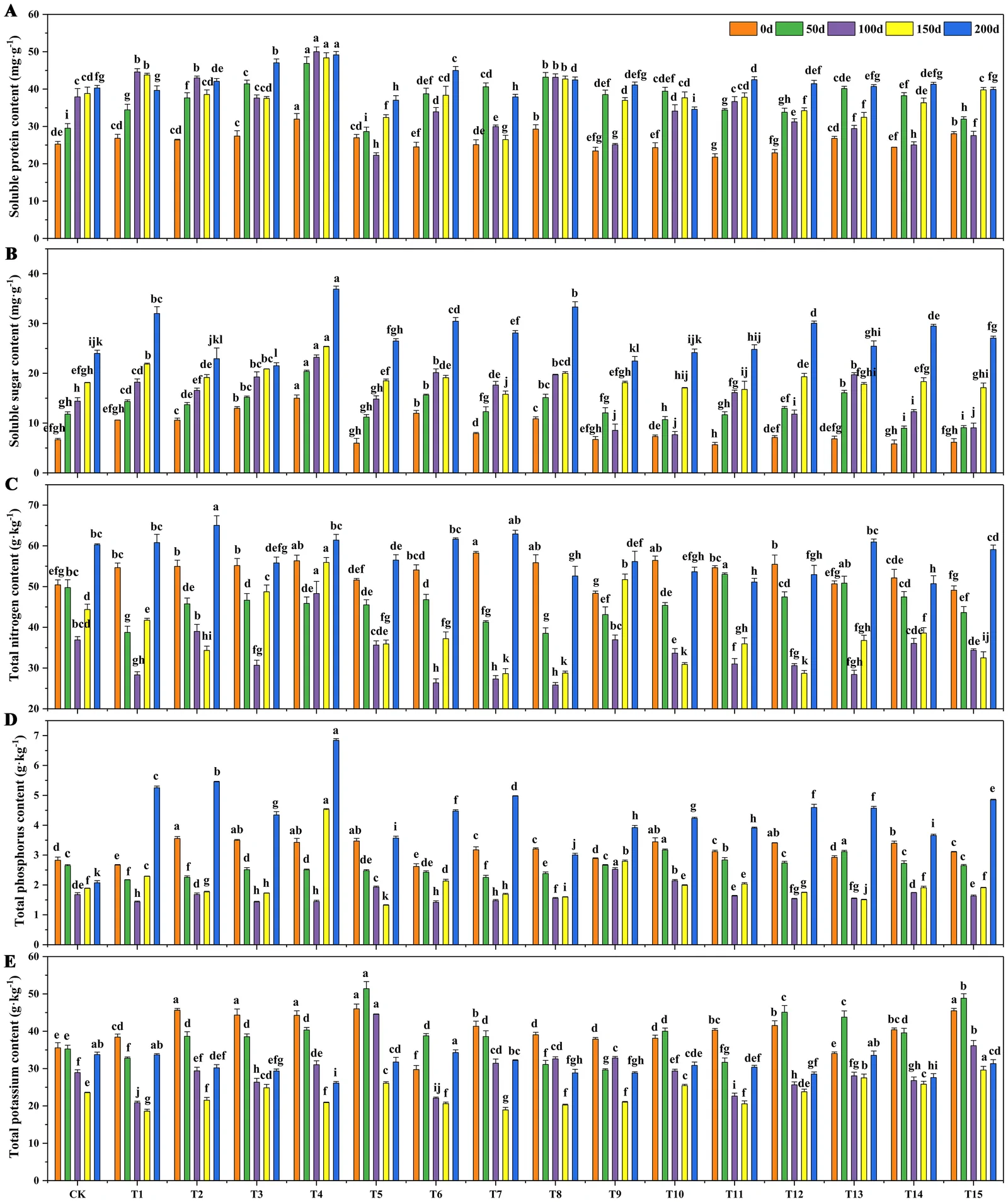
Effects of different substrate treatments on leaf nutrient accumulation of passion fruit (0~200 days). **(A)** Changes in soluble protein content; **(B)** Changes in soluble sugar content; **(C)** Changes in total nitrogen content; **(D)** Changes in total phosphorus content; **(E)** Changes in total potassium content. The error bar indicates the standard errors for three replicates.

The effects of different substrate treatments on leaf nutrient indices of passion fruit varied. Soluble protein content varied inconsistently among treatments: T4 had the highest values (31.96~49.16 mg·g^-1^) across all stages, significantly above others, followed by T8, with both exceeding CK, while T5 remained relatively low overall (**Figure 3A**). Soluble sugar content generally accumulated gradually over the growth period. T4 again showed the highest values (15.00~36.91 mg·g^-1^) across all stages, followed by T1 and T8, with T9 and T15 relatively low, CK, together with T2 and T3, fell into the upper-moderate category (**Figure 3B**). Leaf total N content exhibited a trend of first decreasing and then increasing. At 200 d, T2 had the highest value (65.04 g·kg^-1^), followed by T7, T4, T6, T2, T13 and CK, while T11 and T14 had the lowest (**Figure 3C**). Total phosphorus content also decreased first and then increased. The CK showed only a slight increase in total phosphorus at the late stage, whereas all substrate treatments increased sharply at the late stage, with T4 reaching the maximum value (6.84 g·kg^-1^) at 200 d (**Figure 3D**). Total potassium content was generally higher at the early stage than at the late stage. During 0~100 d, T5 had the highest values (44.56~51.41 g·kg^-1^), followed by T15; at the late stage, the differences among treatments narrowed, and the values generally ranged from 30 to 35 g·kg^-1^ (**Figure 3E**). In summary, different treatments significa -ntly regulated leaf nutrient accumulation and metabolic activity. Among them, T4 showed obvious advantages in the accumulation of soluble protein, soluble sugar, and total phosphorus; T2 performed prominently in total nitrogen; and T5 exhibited the strongest potassium supply capacity at the early stage.

#### 3.2.5 Effects of different substrates on fruit quality and yield of passion fruit

**Figure 4:**
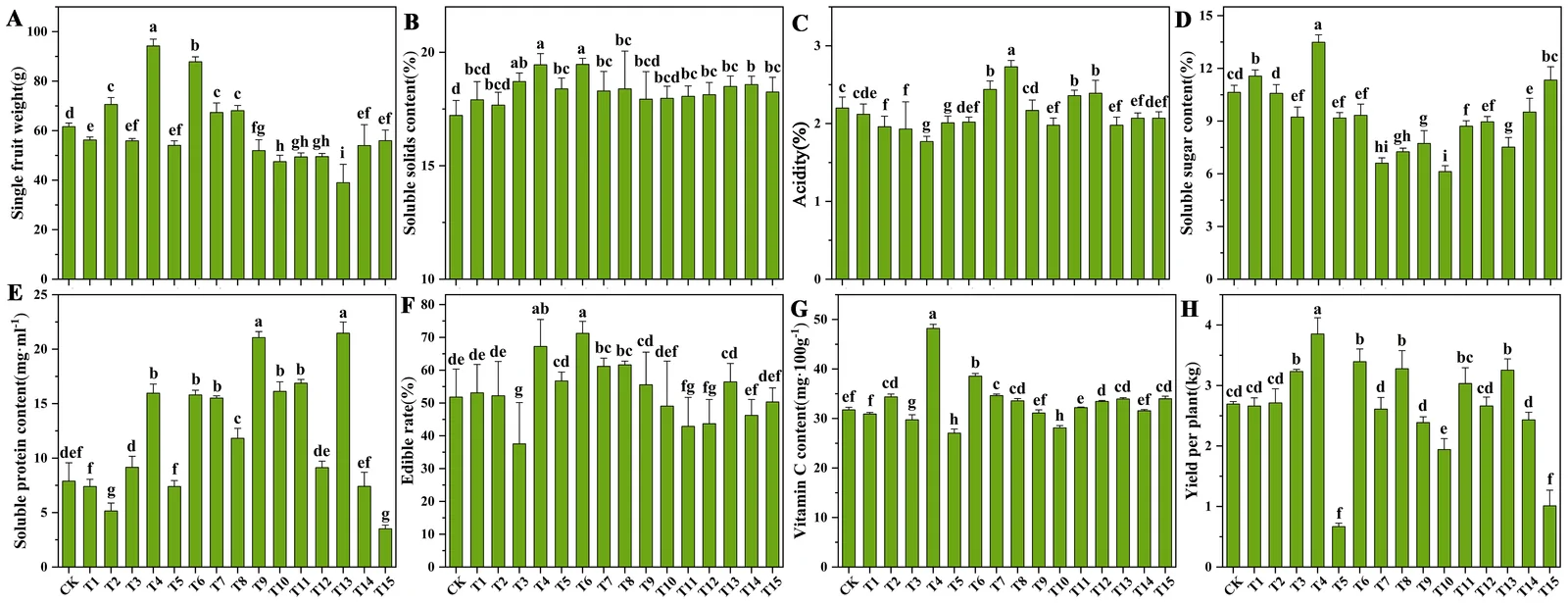
Effects of different substrate formulations on fruit quality and yield in passion fruit. **(A)** Single fruit weight; **(B)** Soluble solids content; **(C)** Acidity; **(D)** Soluble sugar content; **(E)** Soluble protein content; **(F)** Edible rate; **(G)** Vitamin C content; **(H)** Yield per Plant. The error bar indicates the standard errors for ten replicates.

Substrate formulations significantly influenced fruit quality and yield of passion fruit. T4 achieved the highest single fruit weight (94.24 g), soluble solids (19.45%), soluble sugar (13.4%), vitamin C (48.20 mg·100 g^-1^), and yield per plant (3.85 kg), along with relatively high soluble protein (15.96 mg·g^-1^ FW) and edible portion (66.8%); all these indices significantly exceeded CK. It also had the lowest acidity (1.76%), underscoring marked advantages in fruit enlargement, sugar accumulation, nutritional quality, and yield formation. T6 was comparable or second to T4 in single fruit weight, soluble solids, vitamin C, and yield per plant, and gave the highest edible portion (70.5%). The acidity peaked in T8 (2.73%), with some *P. eryngii* SMS-based treatments (T11, T12) also remaining relatively high. Soluble protein content was highest in T13 (21.5 mg·g^-1^ FW), and all *A. heimuer* SMS-based treatments (T7-T10) significantly exceeded the control. In contrast, T3 had the lowest edible portion, while T5 and T15 produced the lowest yields (**Figure 4**, **Supplementary Table 4**). Overall, T4 and T6 markedly improved fruit quality and yield, with T4 showing more comprehensive advantages.

### 3.3 Correlation of substrate properties with plant traits

**Figure 5:**
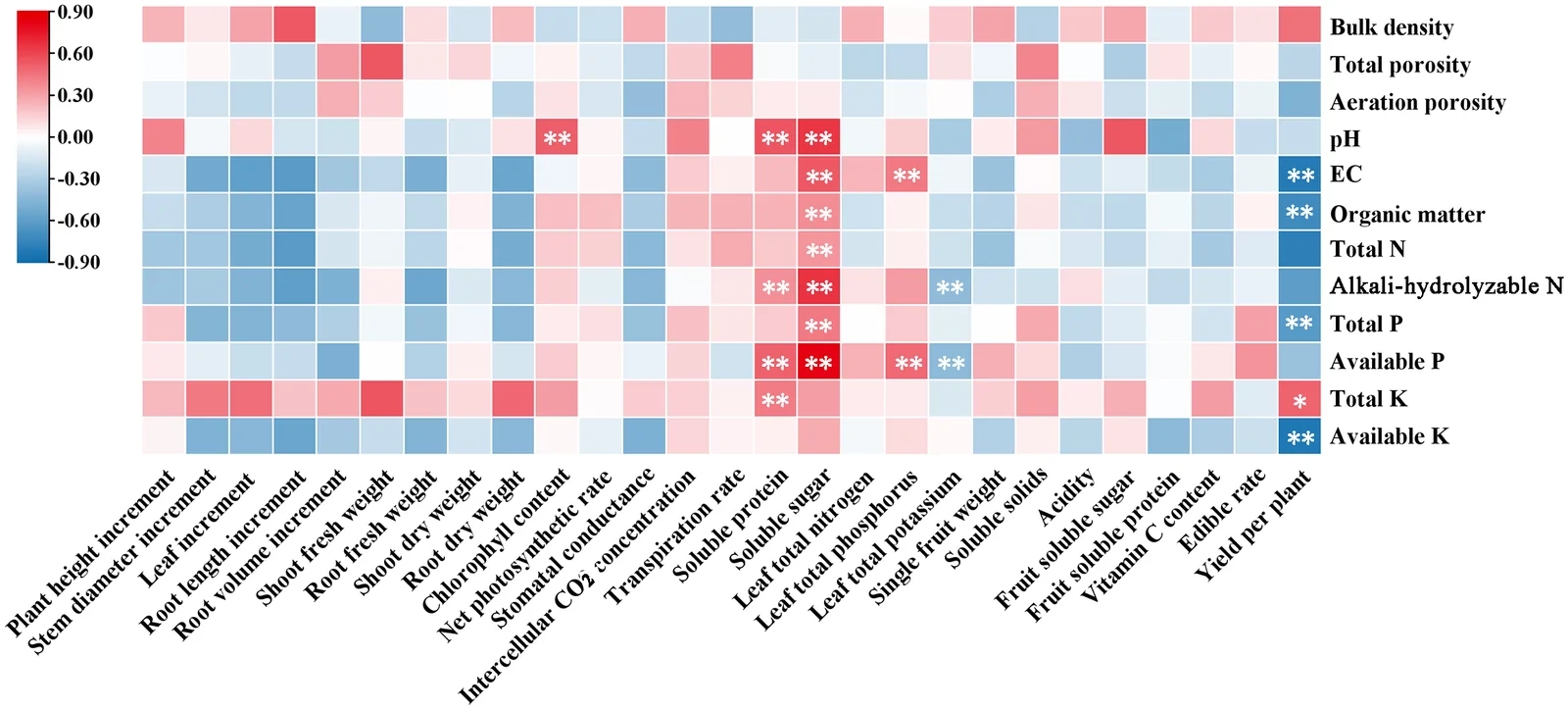
Correlation heatmap between substrate properties and plant traits of passion fruit. Red indicates positive correlations, whereas blue indicates negative correlations. Darker colors represent stronger correlation strengths, and *, ** respectively representing significant correlations at the 0.05, 0.01 levels. Significance is based on FDR-adjusted q < 0.05.

Correlation analysis revealed significant associations between substrate properties and multiple plant parameters in passion fruit, particularly for alkali-hydrolyzable N, EC, pH and available P. Leaf soluble sugar contents exhibited significant to highly significant positive correlations with pH, EC, organic matter, N and P. Similarly, leaf soluble protein was significantly positively correlated with EC, alkali-hydrolyzable N and available P. In contrast, leaf total N showed a significant negative correlation with alkali-hydrolyzable N and available P. Yield per plant was significantly positively correlated with total P only, yet it displayed highly significant negative correlations with EC, organic matter, total K and available K (**Figure 5**). Excessive supply of organic matter, P and K can elevate EC, and high EC inhibits root water and nutrient uptake, thereby reducing yield. Meanwhile, unabsorbed nutrients accumulate in the substrate, resulting in a pattern where high nutrient levels are negatively associated with yield.

### 3.4 Effects of different substrates on rhizosphere microbiota composition of passion fruit

#### 3.4.1 Microbial α- and β-Diversity

Significant differences in α-diversity (Chao1, p=1.13×10^−9^; Shannon, p=6.16×10^−12^) of rhizosphere bacterial and fungal communities of passion fruit were observed among treatments. For bacteria, both Chao1 and Shannon indices in CK were significantly lower than those in other treatments. Substrate mixtures containing mushroom residue generally increased bacterial α-diversity, with T15 showing the highest richness and diversity indices, which were significantly higher than those of other treatments (**Supplementary Figures 4A, B**). For fungi, T4 showed the highest values, with both Chao1 and Shannon indices significantly higher than those of other treatments, followed by T3 and T15, while CK and T6 had relatively lower values. These results indicate that different mushroom residue ratios effectively regulate the compositional complexity of rhizosphere microbial communities (**Supplementary Figures 4C, D**). PCoA ordination and ANOSIM analysis based on Bray-Curtis distance revealed clear separation in the composition of both bacterial and fungal communities among different substrate treatments. Replicates within the same treatment clustered closely, while community structures differed significantly between treatments (**Supplementary Figure 5**).

#### 3.4.2 Characteristics of rhizosphere microbial community composition in passion fruit

**Figure 6:**
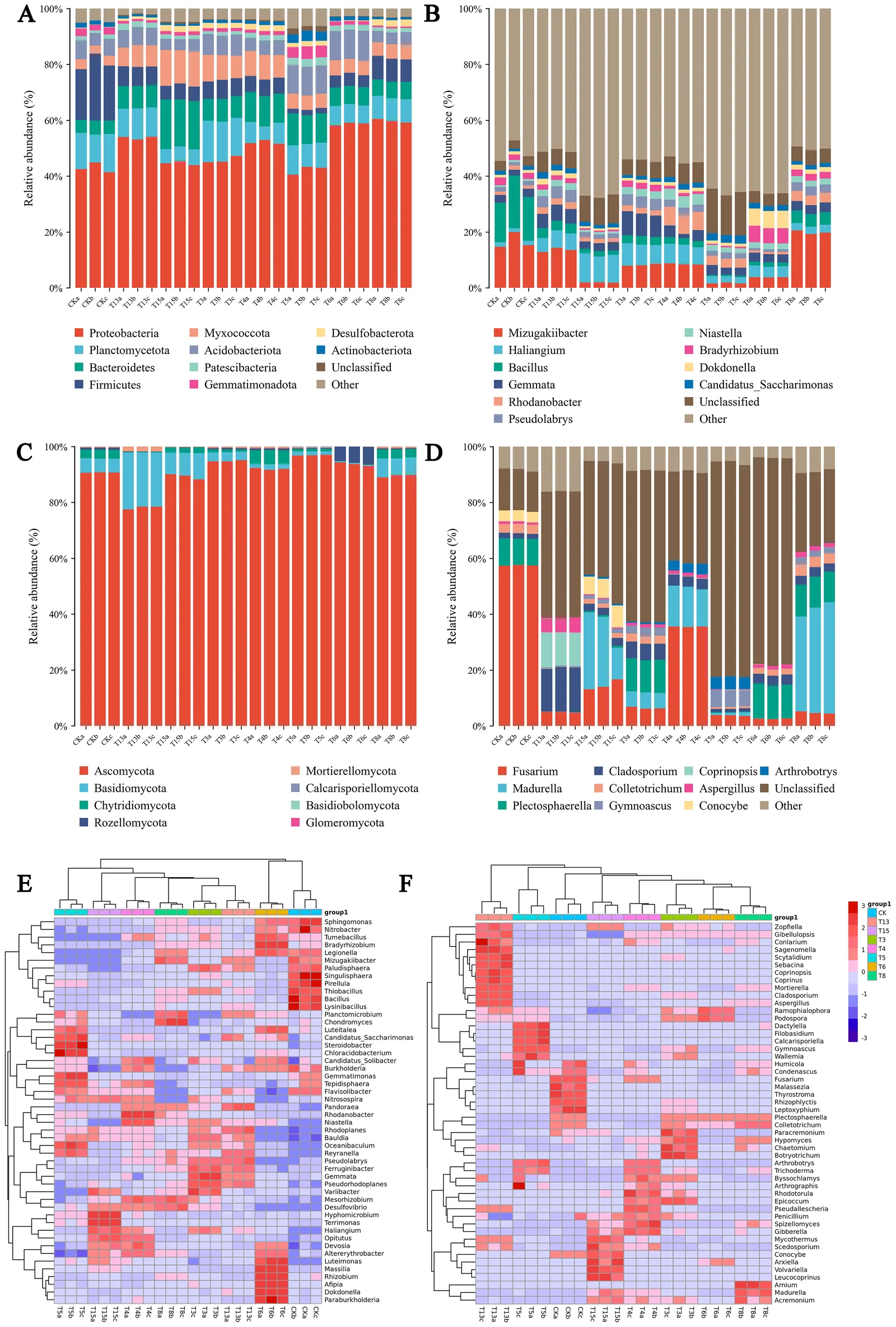
Characteristics of rhizosphere microbial community composition in passion fruit. Bar chart of bacterial community composition at the phylum level **(A)** and the genus level **(B)** in the rhizosphere; Bar chart of fungal community composition at the phylum level **(C)** and the genus level **(D)** in the rhizosphere; Heatmap of genus-level abundance of rhizosphere bacterial **(E)** and fungal **(F)** communities.

The composition of rhizosphere microbial communities of passion fruit differed significantly among substrate formulations. At the phylum level, bacterial and fungal communities were consistently dominated by Proteobacteria (relative abundance 40~60%) and Ascomycota (>80% relative abundance) (**Figures 6A, C**), respectively, but the relative abundances of subdominant phyla and dominant genera varied markedly across treatments (**Figures 6B, D**), with clear separation in the clustering of dominant taxa (**Figures 6E, F**), indicating strong treatment specificity. LEfSe analysis revealed treatment-specific signature microbial taxa. For bacteria, T4 mainly enriched *Niastella* and *Rhodanobacter*; T6 enriched a greater number of differential bacterial taxa; and T15 was characterized by enrichment of *Haliangium* (**Supplementary Figures 6A, C**). For fungi, T13 mainly enriched *Cladosporium*; T6 enriched *Sordariales* and *Plectosphaerella*; and T8 showed notable enrichment of *Madurella* (**Supplementary Figures 6B, D**).

Across all treatments, 29 bacterial phyla and 531 bacterial genera were detected in the rhizosphere soil of passion fruit. At the phylum level, Proteobacteria dominated (42.96~59.82% relative abundance), followed by Planctomycetota, Bacteroidetes, Firmicutes, Myxococcota and Acidobacteriota, and their abundances varied by treatment (**Figure 6A**). At the genus level, the top 10 by average relative abundance were *Mizugakiibacter*, *Haliangium*, *Bacillus*, *Gemmata*, *Rhodanobacter*, *Pseudolabrys*, *Niastella*, *Bradyrhizobium*, *Dokdonella* and *Candidatus Saccharimonas* (**Figure 6B**); these dominant genera mainly fell within the four most abundant phyla. Heatmap analysis revealed strong treatment-specific patterns (**Figure 6E**). CK enriched *Singulisphaera*, *Nitrobacte*r, *Sphingomonas*, *Bradyrhizobium*, *Bacillus* and *Lysinibacillus*, likely due to its loess-based substrate, which is compact, poorly aerated and low in organic matter. T4 enriched *Rhodanobacter*, *Niastella*, *Devosia*, *Nitrosospira* and *Mesorhizobium*. T3 and T5 (both *P. geesteranus* SMS-based) mainly enriched *Variibacter* and *Gemmata* (T3), and *Steroidobacter* and *Gemmatimonas* (T5), respectively. T6 (*A. heimuer* SMS-based) enriched *Bradyrhizobium*, *Dokdonella*, and *Devosia*, whereas T8 (*A. heimuer* SMS-based) had higher abundance of *Chondromyces*. T13 enriched *Pandoraea*, *Gemmata*, and *Niastella*, while T15 notably enriched *Terrimonas* and *Hyphomicrobium*. Overall, the composition of dominant bacterial genera varied substantially across treatments. LEfSe analysis (Kruskal-Wallis test, *p* < 0.05; LDA> 4.0) showed that CK was characterized by the Firmicutes-Bacilli-Bacillales clade; T3 by the Planctomyce -tota-*Gemmata* clade; T4 by *Rhodanobacter* and *Niastella*; T5 by the Gemmatimonadota-*Gemmatimo -nas* clade; and T15 by Bacteroidetes, Myxococcota and *Haliangium*-related groups. T6 enriched the most diverse differential taxa, spanning multiple phylogenetic lineages including *Bradyrhizobium*, *Dokdonella* and Burkholderiales (**Supplementary Figures 6A, C**).

A total of 6 fungal phyla and 150 fungal genera were detected. Ascomycota was the dominant phylum (78.17~96.93% relative abundance), followed by Basidiomycota, Chytridiomycota and Rozellomycota, and their abundances varied across treatments (**Figure 6C**). At the genus level, **Figure 6D** shows significant differences in abundance among the ten dominant genera, with Fusarium, Madurella, Plectosphaerella, and Cladosporium being more abundant. Heatmap analysis revealed distinct differences in dominant genera across treatments. CK enriched *Fusarium*, *Malassezia* and *Thyrostroma*; T3 enriched *Chaetomium* and *Botryotrichum*; T4 enriched *Rhodotorula*, *Pseudallescheria*, *Penicillium* and *Spizellomyces*; T5 enriched *Dactylella*, *Filobasidium* and *Calcarisporiella*; T6 enriched *Ramophialophora* and *Podospora*; T8 enriched *Colletotrichum*, *Arnium* and *Madurella*; T13 enriched *Coprinus*, *Coprinopsis*, *Conlarium* and *Sagenomella*; T15 enriched *Mycothermus*, *Arxiella* and *Volvariella*. LEfSe analysis (Kruskal-Wallis test, *p* < 0.05; LDA> 4.0) identified treatment-specific fungal taxa. CK was enriched in Ascomycota and the *Fusarium*-related clade. T3 (*P. geesteranus* SMS-based) enriched the Chaetomiaceae clade, while T5 enriched Ascomycota, Orbiliales and Gymnoascaceae-related groups. T6 (*A. heimuer* SMS-based) enriched Sordariales and *Plectosphaerell -a*-related groups, while T8 notably enriched *Madurella*-related groups. T13 (*P. eryngii* SMS-based) enriched Basidiomycota and the *Cladosporium*-related clade, and T15 enriched Dothideomycetes and Microascales-related groups (**Supplementary Figures 6B, D**). Thus, substrate composition affects both the overall rhizosphere microbial community structure and selectively shapes specific taxa.

#### 3.4.3 Correlation of microbial community with substrate properties and plant traits

**Figure 7:**
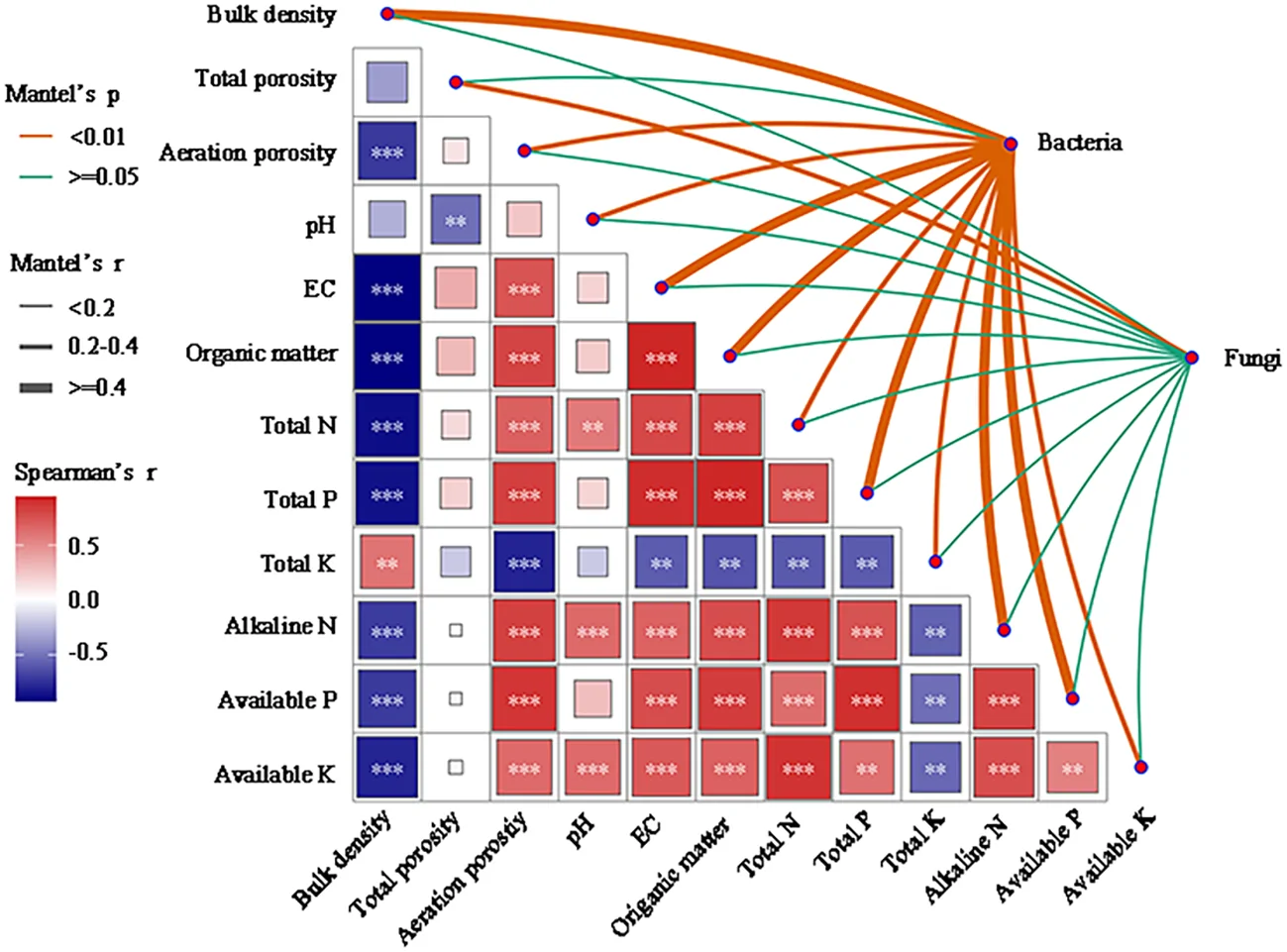
Mantel test and Spearman correlation analysis between substrate physicochemical properties and rhizosphere microbial communities of passion fruit. Rectangular frames indicate the correlations between different physicochemical properties factors established by Pearson, with blue indicating negative correlation and red indicating positive correlation, and the deeper the color, the more significant the correlation. Different ranges of P-values in the figure are represented by lines of different colors, and the Mantel test_r values are indicated by the thickness of the lines, with *, **, *** respectively representing significant correlations at the 0.05, 0.01, 0.001 levels.

Spearman correlation analysis revealed strong coupling among substrate physicochemical properties. Bulk density was significantly negatively correlated with all indicators except total potassium, total porosity and pH, while aeration porosity showed significant positive correlations with most indicators (except bulk density and total K). Nutrient indicators were generally positively intercorrelated, but total K was negatively correlated with all other nutrient indicators. Mantel tests further showed that rhizosphere bacteria correlated more strongly with substrate properties than did fungi (**Figure 7**).

**Figure 8:**
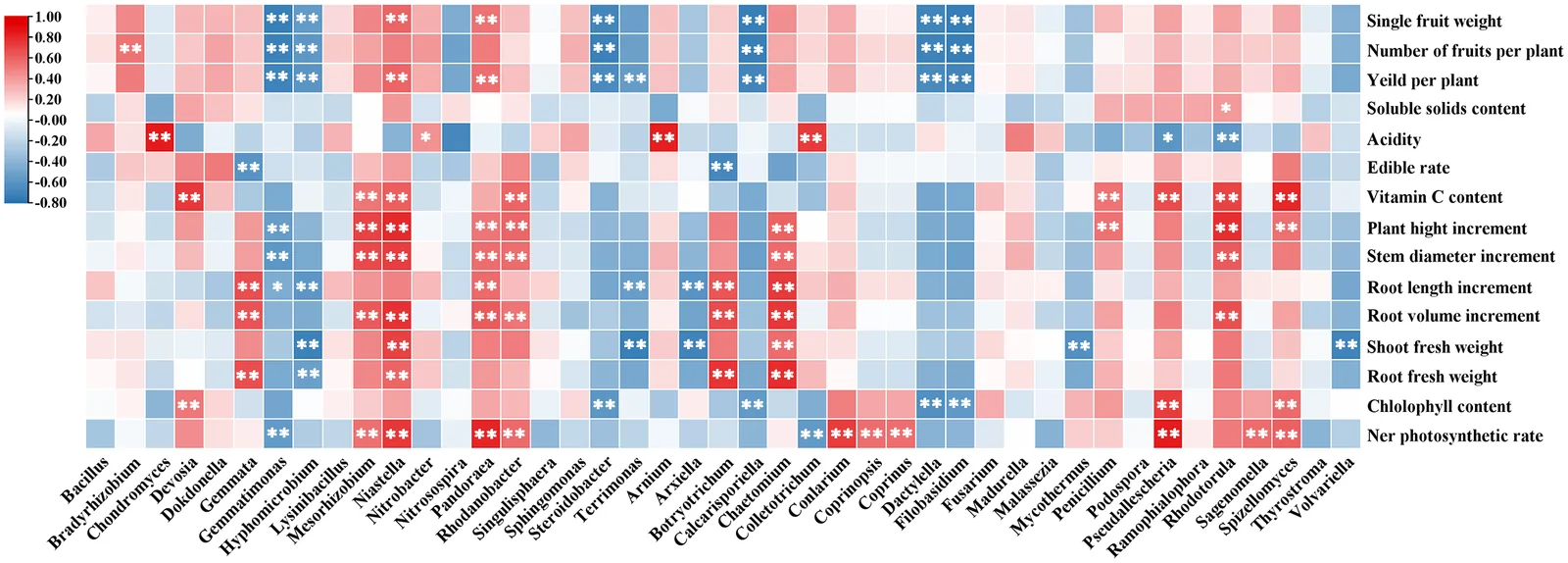
Pearson correlation heatmap between dominant rhizosphere microbial taxa and plant traits of passion fruit. Red indicates positive correlations, whereas blue indicates negative correlations. Darker colors represent stronger correlation strengths, and *, ** respectively representing significant correlations at the 0.05, 0.01 levels. Significance is based on FDR-adjusted q < 0.05.

Spearman correlation analysis was used to assess relationships between dominant rhizosphere microbes and passion fruit growth, yield and quality. *Niastella, Mesorhizobium, Pandoraea* and *Rhodanobacter* showed significantly positive correlations with most vegetative and fruit traits (plant height, stem diameter, root parameters, biomass, photosynthesis, fruit weight, yield, fruit quality parameters). *Gemmata*, *Devosia*, and *Bradyrhizobium* also exhibited positive correlations, with *Gemmata* associated with plant growth indicators, *Devosia* linked to chlorophyll content and photosynthesis, and *Bradyrhizobium* correlated with fruit weight and yield. Among fungi, *Chaetomium*, *Botryotrichum*, *Rhodotorula*, *Pseudallescheria* and *Spizellomyces* were positively correlated with growth and quality. In contrast, the bacterial genera *Gemmatimonas*, *Hyphomicrobium*, *Terrimonas* and *Steroidobacter*, as well as the fungal genera *Dactylella*, *Filobasidium* and *Calcarisporiella*, were negatively correlated with most of the growth and yield parameters (**Figure 8**). Overall, positively correlated taxa (e.g., *Niastella*) may promote vegetative growth and high-quality yield, whereas negatively correlated taxa (e.g., *Gemmatimonas*) may suppress them.

## 4 Discussion

### 4.1 Effects of physicochemical properties of substrates on passion fruit growth and yield

Composting effectively promotes stabilization and substrate utilization of mushroom residues, yet the physicochemical and nutrient properties of composted residues vary markedly among types (Paula et al., 2017; Lou et al., 2017). In this study, composting of *P. eryngii*, *P. geesteranus* and *A. auricula* residues differed due to variations in readily decomposable organic matter and particle structure. Composting raised bulk density and shifted pH toward weak alkalinity, reducing pH variability among residues, but didn’t eliminate inherent differences in pore structure and nutrient supply-consistent with the finding that feedstock composition governs nutrient transformation and humification (Lou et al., 2017; Wang et al., 2024). Mixing composted residues with seaweed mud and river sand generally decreased bulk density and increased total and aeration porosity, corroborating earlier reports that residue substitution improves porosity (He et al., 2024). Despite low bulk density and high porosity, mushroom residues often have high EC and poor nutrient retention (Meng et al., 2018; Quisth et al., 2025; Ma et al., 2025). Soil EC, pH and organic matter interact dynamically, collectively shaping root-zone physicochemical conditions and plant growth (Zuo et al., 2023; Yan et al., 2024). Passion fruit is salt-sensitive, Lima et al. (2023) reported threshold EC values of 0.3~1.0 dS·m^-1^ for three sour cultivars, above which yields dropped markedly (Lima et al., 2023; Souza et al., 2025). In our study, EC was generally higher in substrate-mix treatments than in CK, especially in the single-residue treatments T5, T10, and T15, which reached 6.12~8.24 in later stages and showed pronounced defoliation. EC correlated negatively with yield per plant, likely because elevated EC raises root-zone osmotic pressure, restricting water and nutrient uptake (Rengasamy, 2010; Lu and Fricke, 2023). Optimal pH for passion fruit is 5.5~6.5 (Fan et al., 2025), but Obike et al. (2026) found that yellow-fruited genotypes tolerate pH 4~8, while purple-fruited ones prefer pH≈5 (Cheng et al., 2025; Obike et al., 2026). Here, substrate pH ranged 6.40~8.39, and ‘Qinmi No. 9’ grew and fruited normally under these mildly alkaline conditions, likely due to its broad pH tolerance. Given that passion fruit genotypes differ in salt and pH tolerance, future studies should systematically evaluate the performance of the T4 formulation across a broader range of passion fruit cultivars and rootstock-scion combinations. Soil organic matter (SOM), a key health indicator, increased significantly with residue substrates versus CK. However, SOM correlated negatively with yield, and not positively with fruit quality. Notably, the single-residue treatments had high SOM but lower yields, contrasting with prior reports (Fan et al., 2025; Cheng et al., 2025), likely because of excessive EC, underscoring the complex interplay among EC, pH and SOM.

### 4.2 Effects of substrate ratios on growth and yield of passion fruit

Organic substrate at an appropriate ratio enhances stomatal conductance and transpiration by optimizing root-zone water-air balance, thereby boosting photosynthesis and synergistically improving crop yield and nutritional quality (Sarkar et al., 2021; Aydi et al., 2023). Similarly, alkaline humic acid fertilizers (Fan et al., 2025), mushroom-residue bio-organic fertilizers (Cheng et al., 2025), and organic wastes (Antunes et al., 2022) have been reported to improve passion fruit yield and quality via rhizosphere physicochemical and microbial enhancement. In our study, treatments T3, T4 and T6 outperformed others, with T4 (*P. geesteranus* SMS:seaweed mud:river sand = 3:1:1) showing the best overall performance in both physiological traits (net photosynthesis, stomatal conductance, chlorophyll, soluble sugar and protein content) and yield components (fruit weight, size, soluble sugar, vitamin C, soluble solids, fruit number and yield per plant). This quality advantage may be attributable to two factors: (1) the improved root-zone environment-stable pH, moderate EC, and good aeration, facilitated efficient potassium uptake, essential for sugar translocation and accumulation (Basil et al., 2024); and (2) sustained higher photosynthetic rate and leaf soluble sugar during the reproductive stage indicated enhanced carbon assimilation and source-sink translocation, favoring fruit sugar and dry matter accumulation (Shim et al., 2022). In contrast, the pure residue treatments (T5, T10, T15) had higher organic matter and nutrient contents but lacked the structural regulation provided by seaweed mud and river sand, which led to elevated EC, salt accumulation, and poor root-zone coordination, resulting in lower yield and quality. This confirms that the efficacy of mushroom residues is constrained by feedstock source, processing method, and blending ratio (Chen et al., 2024; Quisth et al., 2025). Thus, optimizing passion fruit substrate formulation should focus not merely on increasing the residue substitution rate, but on constructing a composite system that harmonizes aeration, water retention, and nutrient supply.

### 4.3 Substrate ratios affect passion fruit growth and yield by reshaping the rhizosphere microbiome

Substrate ratios shape rhizosphere microbial communities and thus passion fruit performance, but higher α-diversity alone does not guarantee superiority; rather, a community structure aligned with plant growth and fruiting demands is critical (Wang et al., 2022; Huang et al., 2023; Cheng et al., 2025). In our study, T4, despite not having the highest bacterial α-diversity, was enriched in beneficial taxa (*Niastella*, *Mesorhizobium*, *Rhodanobacter* and *Devosia*) that correlated positively with vegetative growth, yield and quality. *Niastella*, as reported by Cuartero et al. (2022) maintains soil fertility via nitrogen-cycling genes (*nosZ*), and Yu et al. (2024) identified it as a plant growth-promoting bacterium that secretes IAA and gibberellins, enhancing stress tolerance and biomass. Its accumulation in T3, T4, and T13 likely improved the rhizosphere microenvironment, promoting both vegetative and reproductive growth across developmental stages. Moreover, *Mesorhizobium* fixes nitrogen (Liu Z. X. et al., 2022), *Rhodanobacter* possesses denitrification and biocontrol potential (Woo et al., 2024), and *Devosia* enhances growth and nitrogen uptake (Li et al., 2020; Zhang et al., 2024); their co-enrichment in T4 may have contributed to enhanced growth and fruit set. In CK, genera such as *Singulisphaera*, *Nitrobacter*, *Sphingomonas* and *Bradyrhizobium* were enriched, some with known beneficial traits (Shi et al., 2022; Qiao et al., 2024; Zhang et al., 2026), others associated with disease (Zheng et al., 2022), suggesting that the more soil-like rhizosphere tends to favor generalist taxa adapted to basic nutrient cycling and environmental stress. For fungal communities, T4 enriched *Rhodotorula*, *Pseudallescheria*, and *Spizellomyces*, while T3 enriched *Chaetomium* and *Botryotrichum*, all positively correlated with growth. *Rhodotorula* was found to promote plant growth by increasing soil enzyme activity (Silambarasan et al., 2022); *Pseudallescheria* was considered a phosphatesolubilizing fungus that significantly promoted strawberry growth (Fitriatin et al., 2025); *Spizellomyces* in the rhizosphere of *Panax notoginseng* might either be an indicator genus proliferating in response to ginsenoside Rg or be associated with disease occurrence (Bao et al., 2023). The specific enrichment of these taxa may be beneficial to passion fruit growth and fruiting.

In contrast, T5 enriched the bacterium *Gemmatimonas* and the fungi *Dactylella*, *Filobasidium* and *Calcarisporiella*, while T15 enriched *Terrimonas* and *Hyphomicrobium*; these microbial communities generally showed negative correlations with most growth and yield parameters. Yet the literature indicated they often had positive roles: *Gemmatimonas* abundance signaled high nutrients and active metabolism (Liu C. et al., 2022), *Terrimonas* fixed nitrogen (Visioli et al., 2018), *Hyphomicrobium* contributed to soil organic carbon accumulation (Liu et al., 2025), and *Dactylella* trapped nematodes (Ahren et al., 1998). Therefore, the poor performance of T5 and T15 likely arises from the absence of the beneficial consortia seen in T3/T4, rather than merely from the presence of these negatively correlated taxa, compounded by deteriorating substrate physicochemical conditions at later stages. Substrate properties (bulk density, aeration porosity, EC and available nutrients) are known regulators of microbial community structure (Wang et al., 2022; Huang et al., 2023), implying that formulation optimisation drives a physicochemical-microbial-plant synergy (Deng et al., 2022; Yang et al., 2023). T4 achieved a superior balance among aeration, water retention, salt buffering, and nutrient release, while enriching key growth-promoting taxa, resulting in the best overall performance in vegetative growth, fruit quality, and yield. Nevertheless, these associations are correlational, future research should therefore employ cultivation-dependent approaches combined with transcriptomic or metabolomic profiling to establish causal links between specific taxa and plant growth promotion.

## 5 Conclusions

This study evaluated 15 compound substrates from composted spent mushroom substrate (SMS) for passion fruit soilless cultivation. Formulation T4 (*P. geesteranus* SMS:seaweed mud:river sand = 3:1:1) outperformed all others, significantly increasing yield per plant and improving fruit quality. Its success stemmed from balanced physicochemical properties (stable bulk density, moderate EC, adequate aeration) and enrichment of beneficial rhizosphere bacteria positively correlated with growth and quality. Pure SMS formulations, despite high nutrients content, failed due to excessive EC and structural instability, thus future research should explore blending SMS with natural soil to optimize substrate performance. T4 offers a sustainable, effective alternative to soil-based cultivation, supporting SMS valorization and passion fruit production.

## Data Availability

The datasets presented in this study can be found in online repositories. The names of the repository/repositories and accession number(s) can be found below: https://ngdc.cncb.ac.cn/gsa/browse/CRA045236, NGDC.
