# Supplementary material for: Effects of spent mushroom substrate cultivation under greenhouse conditions on growth and fruiting of passion fruit
Source: Front Plant Sci. 2026 Sep 7;17:1926220. doi: 10.3389/fpls.2026.1926220 (PMC13590335; doi:10.3389/fpls.2026.1926220)
Supplement: Supplementary file 1 [file Supplementaryfile1.doc]

**Supplemental Table 1. Temperature variation characteristics during composting of different spent mushroom substrates.**

| SMS Type | Heating period (time) | Maximum temperature (℃) | Thermophilic phase duration (d) | Total composting duration (d) |
| --- | --- | --- | --- | --- |
| *P.geesteranus* | 3 | 56 | 6 | 28 |
| *A.heimuer* | 4 | 54 | 4 | 32 |
| *P.eryngii* | 4 | 58 | 5 | 30 |

**Supplemental Table 2. Physicochemical properties and nutrient content of different spent mushroom substrates before and after composting.**

| Stage | Substrate Type | Bulk density (g·cm^-3^) | Total porosity (%) | Aeration porosity (%) | pH | EC (mS·cm⁻¹) | Organic matter (%) |
| --- | --- | --- | --- | --- | --- | --- | --- |
| Before composting | *P.geesteranus* | 0.23 ± 0.004 a | 63 ± 0.2 a | 42 ± 1.4 a | 8.62 ± 0.01 a | 3.14 ± 0.12 b | 4.51 ± 0.17 ns |
|  | *A.heimuer* | 0.17 ± 0.003 c | 69 ± 5.7 a | 39 ± 2.6 a | 7.54 ± 0.03 b | 1.82 ± 0.05 c | 4.26 ± 0.06 ns |
|  | *P.eryngii* | 0.19 ± 0.004 b | 45 ± 3.2 b | 26 ± 3.2 b | 7.10 ± 0.04 c | 3.73 ± 0.18 a | 4.61 ± 0.26 ns |
|  |  |  |  |  |  |  |  |
| After composting | *P.geesteranus* | 0.35 ± 0.020 a | 58 ± 3.1 b | 36 ± 2.5 b | 8.53 ± 0.09 ns | 5.16 ± 0.30 a | 4.67 ± 0.08 b |
|  | *A.heimuer* | 0.26 ± 0.017 b | 65 ± 3.1 a | 35 ± 0.6 b | 8.56 ± 0.21 ns | 2.07 ± 0.15 c | 5.23 ± 0.15 a |
|  | *P.eryngii* | 0.25 ± 0.010 b | 62 ± 1.7 ab | 40 ± 0.6 a | 8.44 ± 0.07 ns | 3.67± 0.04 b | 4.84 ± 0.18 b |
| Stage | Substrate Type | Total N (g·kg^-1^) | Total P (%) | Total K (g·kg^-1^) | Alkaline N (mg·kg^-1^) | Available P (mg·kg^-1^) | Available K (mg·kg^-1^) |
| Before composting | *P.geesteranus* | 2.17 ± 0.11 a | 0.52 ± 0.05 a | 13.65 ± 1.22 a | 204.57 ± 7.25 b | 237.70 ± 13.41 c | 5930 ± 100 a |
|  | *A.heimuer* | 1.68 ± 0.25 b | 0.39 ± 0.05 b | 11.02 ± 0.36 b | 250.96 ± 23.13 a | 302.73 ± 10.86 b | 1360 ± 220 b |
|  | *P.eryngii* | 2.17 ± 0.14 a | 0.52 ± 0.07 a | 13.29 ± 0.66 a | 246.30 ± 6.47 a | 928.39 ± 15.48 a | 5950 ± 220 a |
|  |  |  |  |  |  |  |  |
| After composting | *P.geesteranus* | 2.10 ± 0.15 ns | 0.68 ± 0.10 a | 13.75 ± 0.76 a | 196.47 ± 36.35 ns | 219.84 ± 13.77 c | 6660 ± 160 a |
|  | *A.heimuer* | 1.77 ± 0.48 ns | 0.43 ± 0.02 b | 11.48 ± 0.25 b | 218.58 ± 16.35 ns | 313.68 ± 22.86 b | 1260 ± 170 c |
|  | *P.eryngii* | 2.29 ± 0.03 ns | 0.49 ± 0.08 b | 12.11 ± 0.53 b | 232.30 ± 15.73 ns | 842.95 ± 55.44 a | 5460 ± 170 b |

Note: Different lowercase letters within the same column indicate significant differences among treatments according to Duncan’s multiple range test (P<0.05); ns indicates no significant difference. The same below.

**Supplemental Table 3. Effects of different substrate treatment on biomass increment of passion fruit (0~50 d).**

| Treatment | Root length increment (mm) | Root volume increment (cm^3^) | Shoot fresh weight (g) | Root fresh weight (g) | Shoot dry weight (g) | Root dry weight (g) |
| --- | --- | --- | --- | --- | --- | --- |
| CK | 92.75 ± 1.86 ab | 62.37 ± 7.70 g | 31.57 ± 16.64 cde | 19.23 ± 1.03 def | 98.77 ± 4.84 de | 8.95 ± 1.40 h |
| T1 | 94.92 ± 2.37 ab | 99.83 ± 7.54 c | 253.29 ± 3.51 fg | 18.12 ± 4.44 fg | 65.83 ± 6.70 e | 8.47 ± 0.46 h |
| T2 | 82.34 ± 2.95 de | 41.97 ± 5.77 h | 333.40 ± 16.91 c | 15.04 ± 0.78 gh | 115.77 ± 48.15 cd | 12.96 ± 0.25 c |
| T3 | 90.22 ± 2.01 bc | 110.57 ± 3.55 a | 394.35 ± 20.73 a | 31.84 ± 1.02 a | 210.03 ± 31.02 a | 19.68 ± 1.47 a |
| T4 | 96.75 ± 1.6 a | 110.37 ± 4.85 ab | 399.79± 9.08 a | 24.51 ± 1.50 b | 155.00 ± 8.14 bc | 15.44 ± 0.47 a |
| T5 | 75.39 ± 4.10 f | 58.43 ± 2.92 g | 250.60 ± 19.51 fg | 13.52 ± 2.31 h | 107.07 ± 1.66 de | 5.45 ± 1.20 i |
| T6 | 96.47 ± 0.67 a | 58.57 ± 2.71 g | 274.97 ± 8.74 ef | 17.58 ± 1.11 fg | 167.20 ± 20.57 ab | 10.75 ± 0.51 def |
| T7 | 92.07 ± 3.86 ab | 90.13 ± 3.71 de | 326.13 ± 35.92 cd | 21.88 ± 1.53 bcde | 115.60 ± 21.68 cd | 8.75 ± 0.17 h |
| T8 | 86.43 ± 0.75 cd | 81.77 ± 0.91 ef | 351.50 ± 5.11 bc | 23.45 ± 1.25 bc | 169.33 ± 11.78 ab | 11.10 ± 0.43 de |
| T9 | 79.90 ± 1.35 ef | 90.34 ± 4.95 de | 418.29 ± 43.11 a | 22.75 ± 2.29 bcd | 166.17 ± 15.27 ab | 8.07 ± 0.73 h |
| T10 | 77.31 ± 1.48 ef | 101.44 ± 2.07 bc | 333.27 ± 26.39 c | 24.31 ± 2.14 bc | 176.74 ± 3.97 ab | 10.31 ± 0.27 efg |
| T11 | 77.07 ± 0.32 ef | 89.96 ± 5.03 e | 334.73 ± 12.65 c | 18.37 ± 1.76 efg | 107.47 ± 18.16 de | 11.96 ± 0.56 cd |
| T12 | 81.47 ± 0.76 de | 99.12 ± 4.02 cd | 383.47 ± 4.73 ab | 20.65 ± 1.57 cdef | 200.57 ± 15.01 a | 10.25 ± 0.32 efg |
| T13 | 82.00 ± 0.51 de | 77.89 ± 2.95 f | 289.34 ± 25.37 def | 17.76 ± 1.32 fg | 199.69 ± 7.97 a | 9.40 ± 0.62 fgh |
| T14 | 80.43 ± 1.66 ef | 77.34 ± 2.52 f | 226.14 ± 11.44 g | 19.27 ± 1.33 def | 96.32 ± 42.02 de | 8.64 ± 0.13 h |
| T15 | 82.49 ± 7.05 de | 53.83 ± 2.45 g | 165.11 ± 11.62 h | 13.38 ± 0.90 h | 105.75 ± 20.35 de | 5.15 ± 0.67 i |

**Supplemental Table 4. Effects of different substrate formulations on fruit quality and yield in passion fruit.**

| Treatment | Fruit longitudinal diameter (mm) | Fruit transverse diameter (mm) | Fruit shape index | Number of fruits per plant | Total yield per 667 m² (kg) |
| --- | --- | --- | --- | --- | --- |
| CK | 56.11 ± 2.00 de | 50.12 ± 0.36 de | 1.12 ± 0.05 abc | 35.00 ± 1.73 de | 965.63 ± 14.51 cd |
| T1 | 58.65 ± 1.27 bcd | 48.59 ± 1.57 ef | 1.21 ± 0.05 a | 35.33 ± 2.31 cde | 953.78 ± 48.22 cd |
| T2 | 55.97 ± 1.25 de | 52.35 ± 1.47 c | 1.07 ± 0.04 ef | 34.33 ± 2.08 e | 972.61 ± 83.36 cd |
| T3 | 55.00 ± 2.05 de | 54.57 ± 1.96 b | 1.01 ± 0.05 ef | 40.33 ± 0.58 ab | 1158.71 ± 16.24 b |
| T4 | 64.15 ± 2.20 a | 56.67 ± 1.01 a | 1.13 ± 0.03 abc | 43.67 ± 3.21 a | 1380.12 ± 96.47 a |
| T5 | 47.57 ± 2.17 f | 46.79 ± 1.18 g | 1.02 ± 0.05 def | 12.33 ± 1.53 g | 239.52 ± 7.35 f |
| T6 | 60.42 ± 1.91 abc | 53.78 ± 0.09 bc | 1.23 ± 0.04 abc | 38.67 ± 2.08 bcd | 1216.39 ± 36.69 b |
| T7 | 55.56 ± 2.94 de | 49.92 ± 1.34 de | 1.11 ± 0.08 bc | 39.33 ± 1.53 bc | 935.66 ± 35.75 d |
| T8 | 62.01 ± 1.82 a | 52.50 ± 1.07 c | 1.18 ± 0.05 ab | 37.67 ± 2.31 bcde | 1174.74 ± 106.73 b |
| T9 | 53.45 ± 3.96 e | 50.24 ± 1.25 d | 1.06 ± 0.07 cde | 38.33 ± 2.08 bcde | 855.87 ± 34.24 d |
| T10 | 53.18 ± 4.09 cde | 48.06 ± 1.35 fg | 1.11 ± 0.07 bcd | 29.67 ± 2.89 f | 959.06 ± 53.67 f |
| T11 | 56.90 ± 4.36 cde | 49.91 ± 1.06 de | 1.14 ± 0.08 abc | 36.67 ± 2.52 bcde | 1087.11 ± 36.79 bc |
| T12 | 57.18 ± 3.02 cde | 49.68 ± 1.51 de | 1.15 ± 0.07 bcd | 37.33 ± 1.53 bcde | 954.37 ± 52.06 cd |
| T13 | 46.38 ± 9.76 f | 48.59 ± 1.30 ef | 0.96 ± 0.22 f | 39.67 ± 1.53 ab | 1166.63 ± 36.92 b |
| T14 | 56.03 ± 3.34 de | 49.21 ± 1.38 def | 1.14 ± 0.06 abc | 40.67 ± 0.58 ab | 871.62 ± 53.79 d |
| T15 | 56.08 ± 1.75 de | 48.81 ± 1.56 def | 1.15 ± 0.04 abc | 15.67 ± 2.52 g | 1362.80 ± 53.25 f |

**Supplemental Figure 1. Temperature variation during the composting process of spent mushroom.**

**Supplemental Figure 2. Changes in nutrient contents of different substrate formulations at different stages from 0 to 200 d. The error bar indicates the standard errors for three replicates. Lowercase letters indicate significant differences among the 16 treatments within the same period by one-factor ANOVA analysis (p < 0.05). The same below.**

**Supplemental Figure 3. Changes in photosynthetic characteristics of different substrate formulations. A: Changes in chlorophyll content of different substrate formulations at different stages from 0 to 200 d; B: Effects of different substrate ratios on net photosynthetic rate at 50 d; C: Effects of different substrate ratios on stomatal conductance at 50 d; D: Effects of different substrate ratios on intercellular CO₂ concentration at 50 d; E: Effects of different substrate ratios on transpiration rate at 50 d.**

**Supplemental Figure 4**. **Bacterial (A, B) and fungal (C, D) taxa α-diversity indices of different substrate formulations on passion fruit cultivated. The error bar indicates the standard errors for three replicates.**

**Supplemental Figure 5. PCoA and ANOSIM of rhizosphere fungal communities of passion fruits based on Bray-Curtis distance.**

**Supplemental Figure 6. Analysis of differential microbial taxa of rhizosphere microbial community in passion fruit. LDA histograms of rhizosphere bacterial (A) and fungal (B) communities in passion fruit; LEfSe cladograms of rhizosphere bacterial (C) and fungal (D) communities.**
